# Desynchronization of Theta-Phase Gamma-Amplitude Coupling during a Mental Arithmetic Task in Children with Attention Deficit/Hyperactivity Disorder

**DOI:** 10.1371/journal.pone.0145288

**Published:** 2016-03-01

**Authors:** Jun Won Kim, Bung-Nyun Kim, Jaewon Lee, Chul Na, Baik Seok Kee, Kyung Joon Min, Doug Hyun Han, Johanna Inhyang Kim, Young Sik Lee

**Affiliations:** 1 Department of Psychiatry, Catholic University of Daegu School of Medicine, Daegu, Republic of Korea; 2 Division of Child and Adolescent Psychiatry, Department of Psychiatry, Seoul National University Hospital, Seoul, Republic of Korea; 3 Korea Institute on Neuromodulation, EasyBrain Center, Seoul, Republic of Korea; 4 Department of Psychiatry, Chung-Ang University, College of Medicine, Seoul, Republic of Korea; Sichuan University, CHINA

## Abstract

**Introduction:**

Theta-phase gamma-amplitude coupling (TGC) measurement has recently received attention as a feasible method of assessing brain functions such as neuronal interactions. The purpose of this electroencephalographic (EEG) study is to understand the mechanisms underlying the deficits in attentional control in children with attention deficit/hyperactivity disorder (ADHD) by comparing the power spectra and TGC at rest and during a mental arithmetic task.

**Methods:**

Nineteen-channel EEGs were recorded from 97 volunteers (including 53 subjects with ADHD) from a camp for hyperactive children under two conditions (rest and task performance). The EEG power spectra and the TGC data were analyzed. Correlation analyses between the Intermediate Visual and Auditory (IVA) continuous performance test (CPT) scores and EEG parameters were performed.

**Results:**

No significant difference in the power spectra was detected between the groups at rest and during task performance. However, TGC was reduced during the arithmetic task in the ADHD group compared with the normal group (F = 16.70, p < 0.001). The TGC values positively correlated with the IVA CPT scores but negatively correlated with theta power.

**Conclusions:**

Our findings suggest that desynchronization of TGC occurred during the arithmetic task in ADHD children. TGC in ADHD children is expected to serve as a promising neurophysiological marker of network deactivation during attention-demanding tasks.

## Introduction

Attention deficit/hyperactivity disorder (ADHD) is a neurodevelopmental disorder that is diagnosed when developmentally inappropriate hyperactivity-impulsivity and inattentiveness symptoms are observed in at least 2 settings before the age of 12 [1]. ADHD is among the most common neurobehavioral problems afflicting children between 6 and 17 years of age [2]. The global prevalence of ADHD among children and adolescents is between 8% and 10% [3, 4]. Given its chronic morbidity and disability, management of ADHD is an important public health concern [5]. Although its etiology remains unclear, abnormalities of the circuit connecting the frontal lobe to subcortical regions, the brain areas involved in attentional control and behavioral inhibition, have been suggested as the neurophysiological etiology of ADHD [6]. Various diagnostic tools are being developed to evaluate this hypothesized etiology.

Quantitative electroencephalography (QEEG), which is used to obtain electrophysiological information about the brain and serves as a biomarker of brain activity, is increasingly recognized as a promising tool. Many studies have assessed the use of QEEG to detect differences in brain activity between children with ADHD and controls. Most of these studies have relied on power spectral analysis of individual channels and frequencies. The most robust and consistent findings are an elevated proportion of slower to faster frequencies in the brain, as reflected by the theta/beta ratio [7–9]. Via Fourier transform analysis, the EEG data are split into power and phase, in which power represents the quantity of a specific frequency. The conventional EEG analysis method was referred to as power spectral analysis because it focused on power. However, this method of analysis does not reflect the phase component of the EEG [10]. It has been recently discovered that phase provides information about the timing of neuronal activity. By integrating this information with that for power, the characteristics of the functional activities of the brain can be more accurately described [11].

Theta-phase gamma-amplitude coupling (TGC) is a type of cross-frequency phase-amplitude coupling (CFPAC) that was first discovered in the rat hippocampus when the gamma power was observed to concentrate in specific phases of the theta rhythm during tasks [12]. A similar phenomenon was subsequently observed in the human neural cortex and confirmed as physiological evidence of signal interaction among neuron clusters [13]. The CFPAC reported by Canolty and colleagues (2006) is of interest to clinicians as a potential neurophysiological measure of frontal-subcortical interactions [13]. In contrast to spectral analysis, CFPAC contains phase information generated from EEG oscillations. According to Sauseng and Klimesch (2008), who conducted several studies on phase, the amplitude of an EEG oscillation is affected by the number of neurons that are activated simultaneously. The phase provides information about the timing of neuronal activity [14]. Thus, amplitude and phase provide different types of information about neuronal activity. The complex frontal-subcortical interaction becomes active under conditions of cross-frequency coupling between slow and fast oscillations [14, 15]. An association between frontal-subcortical interaction and coupling has been confirmed in subjects exhibiting anxiety and obsessive symptoms [16, 17]. A study of healthy participants revealed significant associations between short-term memory [18], visual perception [19], learning [20] and decision-making tasks [21].

However, the mechanisms underlying TGC remain unknown, and further investigations are needed to clarify this phenomenon. In particular, there have been limited studies of patients with psychiatric symptom. The aim of our study was to investigate TGC and EEG power spectra during a resting state and during the performance of mental arithmetic tasks in ADHD patients. In previous studies, cross-frequency coupling was found to be more prominent during tasks using working memory compared to during a resting state [22]. Furthermore, a strong association was detected between the short-term memory required for mental arithmetic and cross-frequency coupling, especially with respect to the theta and gamma oscillations [23]. Therefore, we hypothesized that during mental arithmetic tasks, there would be a significant change in cross-frequency coupling and that there would be a difference in TGC between ADHD patients and normal controls. In addition, we investigated the association between the performance of the continuous performance task (CPT), a useful diagnostic tool for ADHD, and EEG analysis results.

## Materials and Methods

### 2.1 Subjects

The subjects were elementary students participating in the “Touch Brain Attention Enhancement Camp” sponsored by Gongju National Hospital from 2011 to 2013. Detailed information about the camp and study was provided to parents and children. Written consent for the medical use of test results and participation of the children in this study was received from the parents of all participants. In addition, after receiving a detailed explanation of the study, all children participated voluntarily and provided written consent for participation. The Gongju National Hospital Clinical Research Ethics Committee approved the content and ethics of this study. The ADHD diagnosis was based on a Korean version of the Diagnostic Interview Schedule for Children Version IV (DISC-IV), which is a structured interview tool, and these diagnoses were confirmed by more than one child and adolescent psychiatrist. We excluded children who were unable to join the camp due to severe ADHD symptoms or were unable to undergo at least 2 minutes of EEG monitoring, despite consent to participation.

In the ADHD group, children who were diagnosed with ADHD based on the DISC-IV criteria were participating in the camp. With respect to the control group, children were included who exhibited no abnormalities based on the DISC-IV criteria and who had no personal history of any psychological disorder or accompanying disease. Children with brain damage, a neurological disorder, a genetic disorder, substance dependence, epilepsy or any other mental disorder reported during a personal history and anamnesis were excluded from participation in this study. Children who exhibited an IQ of 70 or lower according to the Korean-Wechsler Intelligence Scale for Children (Fourth Edition; k-WISC-IV) or who were receiving drug treatment were also excluded from this study.

### 2.2 Experimental procedures

Each subject was seated in a comfortable chair in a dimly lit room and was instructed to relax and keep his or her movements to a minimum. The experiment consisted of two conditions: (1) eyes-closed resting state (control) and (2) mental arithmetic task performance (active). During the mental arithmetic task, the subjects were asked to serially subtract 7 from 1000 as fast as possible with their eyes closed. The task was performed purely mentally to avoid movement-related artifacts. The performance of this task was not controlled during the EEG recordings. We provided the following instructions. "For the next 4 minutes, please keep subtracting 7 from your previous answer starting from 1000. For example, if you keep subtracting 7 from 1000, your answers will be 993, 986, 979, etc. Perform these calculations in your head without using any tools, and tell me your final answer.” However, prior to the experiments, as in previous studies using covert mental arithmetic, practice sessions (serial subtractions) were performed [24]. As a general rule, previous studies have suggested that the evaluation of EEG spectral power in clinical and pharmacological studies should be performed using EEG recordings of at least 120 seconds [25]. After the EEG experiment, the Integrated Visual and Auditory (IVA) IVA CPT was administered to investigate the severity of ADHD symptoms (i.e., hyperactivity-impulsiveness and attentional deficit).

### 2.3 EEG recording and pre-processing

The EEG recordings were performed using a SynAmps2 direct-current (DC) amplifier and a 10–20 layout 64-channel Quick-Cap electrode placement system (Neuroscan Inc., NC, USA). The EEG data were digitally recorded from 21 gold cup electrodes placed according to the international 10–20 system. The impedances were maintained below 5 kΩ, and the sampling rate was 1000 Hz. We used the linked mastoid reference and two additional bipolar electrodes to measure the horizontal and vertical eye movements.

We used Matlab 7.0.1 (Math Works, Natick, MA, USA) and the EEGLAB toolbox [26] to pre-process and analyze the EEG recordings. First, the EEG data were downsampled to 250 Hz. Next, the EEG data were detrended and mean-subtracted to remove the DC component. A 1-Hz high-pass filter and a 60-Hz notch filter were applied to remove the eye and electrical noise. Next, independent component analysis (ICA) was performed to remove the well-defined sources of artifacts. ICA has been demonstrated to reliably isolate artifacts caused by eye and muscle movements and heart noise [27]. Components that corresponded to eye blinking or muscle movement were identified using a published technique that compares favorably with other artifact rejection techniques [28]. We identified and removed at least one component that corresponded to muscle artifacts, and no detected residual muscle artifacts related to ADHD symptom remained in the data. Finally, clinical psychiatrists and EEG experts visually inspected the corrected EEGs. For the analysis, we selected more than two minutes of artifact-free EEG readings from the three-minute recordings.

### 2.4 Power-spectrum analysis of the EEG recordings

Five frequency bands were defined for further analysis: delta (1–4 Hz), theta (4–8 Hz), slow alpha (8–10 Hz), fast alpha (10–13.5 Hz), and beta (13.5–30 Hz). The spectral power of the EEG data was calculated via fast Fourier transformation using the “spectrogram.m” function of the signal processing toolbox in Matlab. Time windows of 1000-ms with an 800-ms overlap and the Hamming window were used for the spectral analysis. Finally, the absolute powers were averaged over all of the time windows and frequency bands for further analysis.

### 2.5 TGC analysis

We used the synchronization index (SI) proposed by Cohen [29] to assess the cross-frequency interactions between the low-frequency (4–8 Hz) phase and the power of the gamma (39–41 Hz) oscillations. Briefly, the SI is a phase coherence measurement between the phase of the upper (gamma) power time series and the phase of the lower time series ($\text{SI}=\frac{1}{\text{n}}\;\sum_{\text{t}=0}^{\text{n}}\;{\text{e}}^{\text{i}}\left[\phi_{\text{lt}}-\phi_{\text{ut}}\right]$; n, the number of time points; ϕ_ut_, the phase of the upper frequency power time series at time point t; ϕ_lt_, the phase of the lower frequency time series at time point t) [29]. To avoid distortions of the phase value during filtering, we used the two-way least-squares finite impulse response filter (eegfilt.m) included in the EEGLAB Toolbox [26]. In addition, 1,000-ms time windows with an 800-ms overlap were used for subject recordings. The gamma power time series was extracted as the squared magnitude of ζ(t), which is the analytic signal obtained from the Hilbert transform (power time series: p(t) = real[ζ(t)]^2^ + imag[ζ(t)]^2^). The phases of the two time series were extracted from the Hilbert transform ($\text{phase}=\text{arctan}\;\left(\frac{\text{imag}\left[\zeta \left(\text{t}\right)\right]}{\text{real}\left[\zeta \left(\text{t}\right)\right]}\right)$). The SI value is a complex number; its magnitude (SIm) reflects the extent to which the phases are synchronized (0 = completely desynchronized; 1 = perfectly synchronized). Outliers were removed in the same manner as described for the spectral analysis. Finally, the SIm values of TGC were averaged over all of the time windows.

### 2.6 Statistical analysis

The clinical and demographic characteristics of both groups (normal controls and ADHD) were analyzed using independent t-tests and chi-square tests. The paired t-test was used to assess the differences between the resting state and the mental arithmetic task performance state. To control for false positives resulting from multiple comparisons, we used the Bonferroni correction, in which the p-values were multiplied by the number of comparisons. To adjust for sex, age and differences in the baseline EEG, the analysis of covariance test (ANCOVA) was performed to compare the differences between the two conditions in each frequency band and TGC between the groups. To improve the clarity of the results, topographical plots of the results of the paired t-test are presented. To assess the relationship between the measures of IVA CPT and the EEG recordings, we used a Pearson’s partial correlation analysis that controlled for sex and age. All data were analyzed using the Statistical Package for the Social Sciences (SPSS) statistical software, version 18.0 (SPSS, Inc., Chicago, IL, USA).

## Results

### 3.1 Demographic characteristics and the IVA CPT scores

There were 53 children (46 males, 7 females) in the ADHD group and 44 children (27 males, 17 females) in the control group. There was a significant difference in the sex ratio between the two groups (χ2 = 8.349, p = 0.004). The ADHD group exhibited significantly lower scores for attention quotient (AQ) and response control quotient (RCQ) (t = 3.096, p = 0.003, and 4.446, p < 0.001, respectively) (Table 1).

**Table 1:** Demographic characteristics and IVA CPT values.

Mean ± S.D.	Control (N = 44)	ADHD (N = 53)	χ2/t	*p*
	N (%)	N (%)		
Age	10.16 ± 1.90	9.62 ± 1.72	1.46	0.149
Sex			8.349	0.004*
Male	27(61.4)	46(86.8)		
Female	17(38.6)	7(13.2)		
IVA CPT				
Attention Quotient (AQ)	100.18 ± 21.01	84.40 ± 29.01	3.096	0.003*
Response Control Quotient (RCQ)	101.82 ± 17.58	82.70 ± 24.66	4.446	< 0.001*

* *p* ≤ .05

SD, Standard deviation; IVA CPT, Intermediate Visual and Auditory Continuous Performance Test

### 3.2 Comparison between groups: resting state and power spectrum

When comparing the mean absolute power value for every frequency band between the groups, significant differences were observed only in the delta frequency band (p = 0.025). No differences were found in the power spectra results at the other frequencies, including theta, slow alpha, fast alpha, and beta, between the groups (all p > 0.05)

### 3.3 Comparison between conditions: paired t-test and ANCOVA

**Fig 1:**
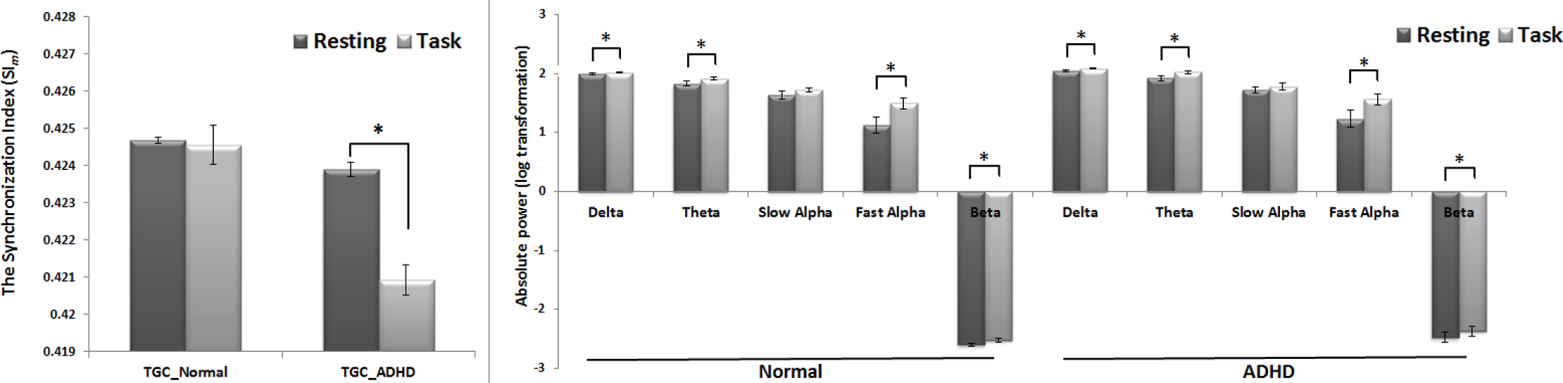
Cross-frequency phase-amplitude coupling and power-spectrum analysis during a mental arithmetic task. The data are reported as the mean ± the standard error of the mean. ADHD, Attention Deficit/Hyperactivity Disorder; TGC, theta-gamma coupling.

**Fig 2:**
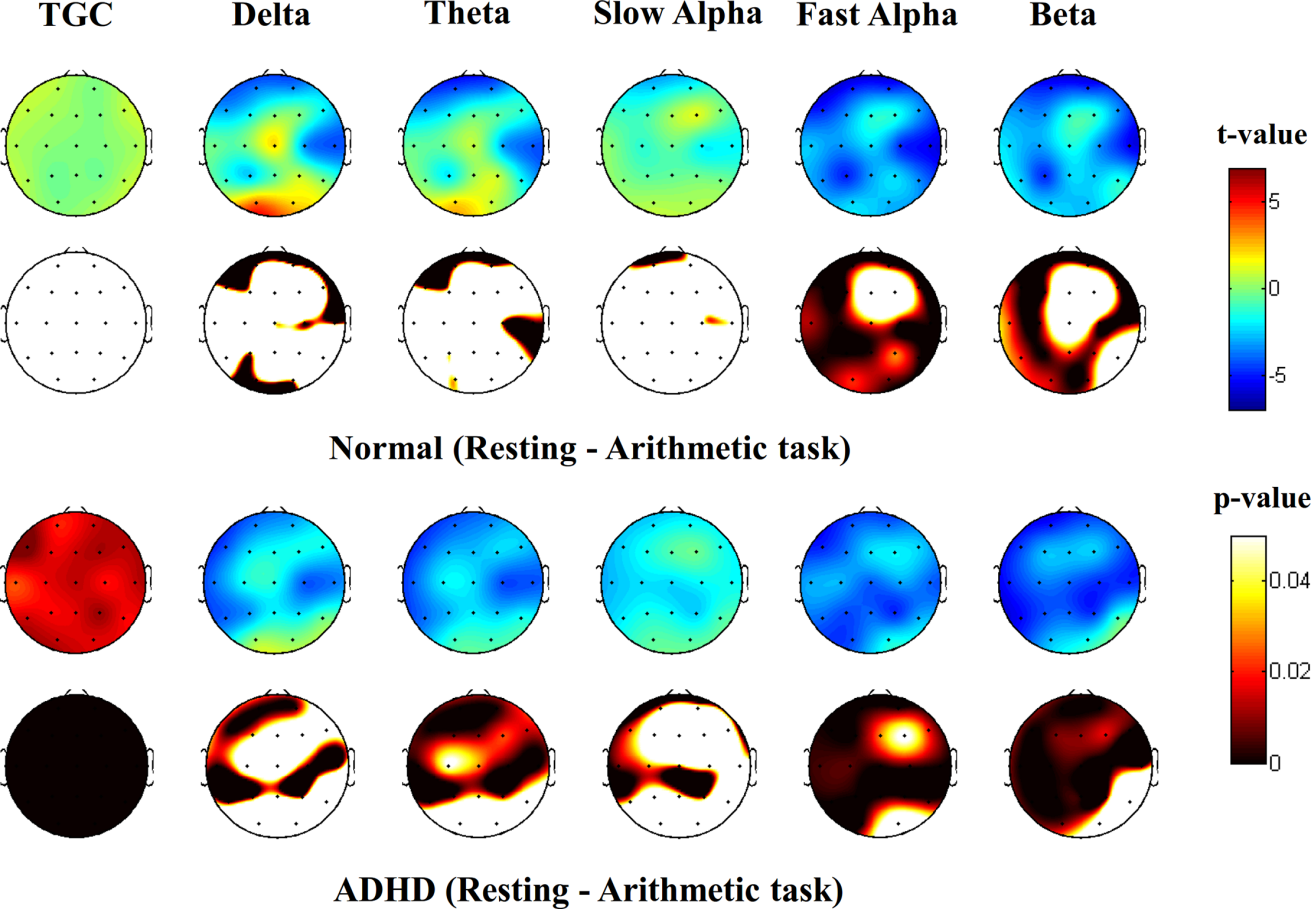
Topographic map of theta-gamma coupling (TGC). The upper topography denotes the topographical distribution of t-values (paired t-test). The lower topography denotes the topographical distribution of the corresponding p-values. ADHD, Attention Deficit/Hyperactivity Disorder.

When comparing the mean absolute power value for every frequency band between conditions, significant differences were observed in all frequency bands except for slow alpha. To determine whether the changes during the arithmetic task were group-dependent and to control for sex, age and differences in the baseline EEG, we compared the differences (resting–arithmetic task performance) between the groups using ANCOVA. No significant differences in the power spectra were detected. However, TGC was reduced during the arithmetic task in the ADHD group compared with the normal group (F = 17.678, p < 0.001) (Table 2 and Fig 1). TGC was further investigated via topographic analysis. The results showed a decrease in TGC in diffuse cortical regions during the arithmetic task in the ADHD group (all p < 0.001) (Fig 2).

**Table 2:** Sex-adjusted means of the differences during the arithmetic task between patients with ADHD and normal controls.

Mean ± S.D.	Differences (Task–Resting)		F	*p*
	Control (N = 44)	ADHD (N = 53)		
TGC*	-0.000057 ± 0.001717	-0.001489 ± 0.001572	16.6963	< 0.001
Delta	0.015083 ± 0.036521	0.021003 ± 0.044434	0.2514	0.617
Theta	0.041091 ± 0.106768	0.052341 ± 0.116621	0.0906	0.764
Slow Alpha	0.041946 ± 0.185929	0.029642 ± 0.190383	0.7775	0.380
Fast Alpha	0.184245 ± 0.370001	0.168342 ± 0.370458	0.4575	0.500
Beta	0.036842 ± 0.043813	0.054810 ± 0.075279	1.3592	0.247

Note: Statistically significant by analysis of covariance

* *p* ≤ .05/6.

The p-value was adjusted using the Bonferroni correction; SD, Standard deviation; ADHD, Attention Deficit/Hyperactivity Disorder; TGC, theta-gamma coupling

### 3.4 Correlation between the IVA CPT scores and the EEG parameters

The results of Pearson’s partial correlation analysis controlled for sex and age between the IVA CPT scores and the EEG parameters are presented in Table 3. TGC positively correlated with the AQ and the RCQ on the IVA CPT. The theta and delta power negatively correlated with the AQ and the RCQ on the IVA CPT. After controlling for the false discovery rate using the Bonferroni correction, the partial correlation between TGC and the theta power remained significant, but the correlation between TGC and delta power did not (Table 3).

**Table 3:** The results of Pearson’s partial correlation analysis (adjusted for sex) of IVA CPT values and EEG analysis.

	AQ	RCQ	Delta	Theta	Slow Alpha	Fast Alpha	Beta	TGC
**AQ**	1.000							
**RCQ**	.768	1.000						
**Delta**	-.235	-.230	1.000					
**Theta**	-.326*	-.325*	.901*	1.000				
**Slow Alpha**	-.153	-.136	.648*	.741*	1.000			
**Fast Alpha**	.055	-.083	.322*	.362*	.537*	1.000		
**Beta**	-.096	-.103	.664*	.680*	.580*	.608*	1.000	
**TGC**	.302*	.286*	-.065	-.134	-.108	.036	0.003	1.000

Note: Significant correlations are indicated in bold.

* *p* ≤ .05/8.

The p-value was adjusted using the Bonferroni correction; IVA CPT, Intermediate Visual and Auditory Continuous Performance Test; EEG, electroencephalogram; AQ, Attention Quotient; RCQ, Response Control Quotient; TGC, theta-gamma coupling

## Discussion

Resting-state power spectra analysis indicated that the only significant difference between the two groups was the delta power. Consistent with the results of previous studies, the ADHD group exhibited a higher delta power [30]. Increase in the delta and theta powers in ADHD have been commonly and highly reliably observed in previous QEEG studies. Further, the delta power increased primarily in the frontal lobe. Similar results have been reported in previous electrophysiological EEG studies of ADHD. This phenomenon is also a component of the maturation lag [31], cortical hypoarousal [32], and developmental deviation theories [33] regarding the developmental aspects of ADHD. An increase in delta power is strongly associated with maturation lag of the brain [31]. The maturation lag theory is supported by studies that have reported similar cortical activity in ADHD children and younger normal controls [34]. ADHD children exhibit a brain maturation lag of approximately 3 years compared to normal peers. This maturation lag displays a different pattern depending on the cortical area involved, and prefrontal cortex maturation can be delayed by a maximum of 5 years [35]. Several researchers have interpreted the increased slow wave activity in children and adolescents with ADHD during an eyes-closed resting condition as evidence of a maturation lag [11, 32].

The mental arithmetic calculation used in this study is often used as an attention-demanding task [36, 37]. Mental arithmetic contains operations that are usually taught in school, such as addition, subtraction, multiplication, and division. Based on the educational curriculum of Korea, we expected that the participants of this study might experience difficulty with multiplication and division. We also expected that because addition is the simplest form of calculation, addition tasks would not provide sufficient cognitive loading for our purposes. Therefore, we selected subtraction as the method of calculation to focus on the attentional control system. Subtraction requires procedural strategies such as decomposing the problem and calculation; these strategies are strongly associated with the posterior parietal cortex, including the intraparietal sulcus [38]. A mental arithmetic task was used to avoid movement-related artifacts during the EEG recordings [24, 31]. In addition, the participants underwent practice trials prior to the evaluation, and we calculated the accuracy of the calculations by evaluating the final calculation results. The arithmetic proficiency of each individual was assessed to determine whether he or she could adequately manage the task. Several practice trials confirmed that all participants were capable of task completion, and we also monitored task performance by reviewing the final calculation results in the actual trial.

In this study, no difference was detected between the groups in the specific frequency bands, as noted above, but a difference in TGC was detected between the groups. This result suggests that ADHD children utilize decreased interactions of the functional neuronal system, such as frontal-subcortical interactions, during the calculation process compared to normal controls. A relationship between attention and gamma activity has been reported previously. In a previous event-related electrophysiological study of attention and gamma activity, an increase in evoked 40 Hz gamma activity was detected during an attention-requiring task compared to at rest. The authors concluded that this evoked gamma activity plays an essential role in top-down attentional processing [39]. To understand the lack of difference between the groups in TGC before the task, we must consider the ‘default-mode network (DMN)’. Marcus Raichle first used this term to explain resting state brain function [40]. Due to the activation of 'task-negative' DMN during the resting state, various areas in the brain exhibit neuronal interaction, as apparently reflected in TGC. The brain regions related to DMN include the precuneus/posterior cingulate cortex (PCC), the medial prefrontal cortex (MPFC) and the medial, lateral and inferior parietal cortices. This network is active in the resting brain with a high degree of functional connectivity between regions but is not actively involved in attention-demanding or goal-directed tasks [40]. When the mental calculation task requiring attention was given, the 'task-negative' DMN was deactivated, and network shifting occurred to activate the 'task-positive' network. However, this network shifting did not occur in the ADHD group of our study, and only deactivation of DMN was observed. Frontal DMN regions are deactivated in children with ADHD when the working memory load increases [41]. Numerous imaging and neurophysiological studies assessing the network in ADHD have been reported, and most have focused on brain activation during cognitive or executive tasks. These studies have revealed deficits in the activity of cortico-subcortical systems that are critical for sustained attention and goal-directed behavior [42–44]. The “task-positive” network that is activated during the cognitive process includes not one but several attentional networks. These attentional networks include the dorsal attention network (DAN), which is related to sustained attention and working memory, and the ventral attentional network, which is associated with attention shifting [45, 46]. To confirm this postulated abnormality in attentional shifting, EEG source reconstruction is required but is not possible with the relatively small number of 19 channels. To compensate for this limitation, previous studies have combined EEG with MRI [47, 48]. Simultaneous EEG-fMRI has been validated as a tool for examining how changes in neuronal oscillations are linked to functional interactions within and between brain networks [48, 49]. However, these studies have been mostly confined to conventional band-limited EEG power, and future simultaneous EEG-fMRI studies including the TGC concept will provide insights on the dynamic functional interactions between brain networks. Despite the lack of specificity, considering the high sensitivity of TGC compared to other EEG parameters, TGC is a promising neurophysiological marker of network deactivation during attention-demanding tasks in children with ADHD. Because this study is the first to report this association, further replicative studies are required to establish the validity of TGC as a biomarker.

We also investigated the association between the EEG parameters and the IVA CPT scores, which correspond to the severity of inattentiveness and hyperactivity/impulsivity in ADHD children. Two characteristics, response control and attention, are differentially involved in various ADHD subtypes [50]. As expected, we demonstrated that the severity of ADHD symptoms significantly correlated with TGC at rest. Based on the power spectral analysis, only theta power displayed a significant association with the CPT scores. The AQ and RCQ demonstrated equally positive correlations with TGC, indicating that the stronger the synchronization, the better the performance exhibited by the children on the CPT. Supposedly, attentional and response control are equally associated with the level of interaction within the attention/arousal systems of the brain. The results of a previous study demonstrating that both working memory and attention are positively correlated with TGC support our results [51, 52].

This study is subject to several limitations. First, no data were collected regarding the presence of any medical condition that could affect the EEG results. In addition, the information about psychiatric status included autism tendency, personality, depression and anxiety. Many studies have reported an association between these factors and specific frequency oscillations [53, 54]. Second, there was a significant difference in the sex ratio between the two groups in our study. Previous studies have excluded girls or included relatively low numbers of girls in research samples and failed to control for possible gender effects [55]. This study could not overcome the limitations of previous results, making it difficult to generalize our results to the whole ADHD population. However, we plan to recruit more girls and compare girls with ADHD and girls without ADHD as well as boys with ADHD and boys without ADHD.

## Conclusion

Quantitative analysis of EEG data is a very informative tool for the diagnosis and treatment of ADHD. However, previous studies of EEG changes in ADHD have been focused on the absolute EEG frequency power or the ratio of the power between different frequency bands. These types of analysis are useful for understanding the neural correlates of ADHD but do not reflect the functional interaction of brain networks. As TGC includes phase, which contains information about neuronal interactions from the EEG recording, TGC is expected to be useful for understanding the mechanisms underlying the deficits in attentional control in ADHD children. In summary, this study investigated top-down attentional control in ADHD children using the novel, evidence-based, neurophysiological measure TGC. The present study suggests that TGC is a promising neurophysiological marker of the attention/arousal system in the brain. Further research exploring the association between TGC and medical or psychiatric conditions that may confound EEG signals will help clarify the potential utility of TGC.

## Supporting Information

S1 Dataset The additional file ‘S1_Dataset.zip’ contains data. In this zip-compressed file, we provide one files: QEEG_CFC_Resting_1007.sav.
(ZIP)
